# A Case of Paralysis

**Published:** 1886-11-27

**Authors:** 


### Difficult Cases.

*The intention of this column is to afford assistance to clergymen, district visitors, hospital officials, medical men, and others who have to provide for the treatment or accommodation of difficult cases of disease, sometimes in its acute, but usually in its chronic form. Anyone being in doubt as to the best course to pursue in any such case, is invited to communicate with the Editor. All such communications should be written on one side of the paper only, and be addressed to the Editor, the envelope being marked in the top left-hand corner, "Difficult Cases."*

### A Case of Paralysis.

Miss Eleanor D. Thompson writes from 44, Porchester Square, W:—"I am seeking a home for a poor lady who two years since was paralysed to the extent of losing her hearing, speech, and the use of her hands, the lower limbs being also affected. For many years she has been an inmate of 'The Ladies' Home,' 53, Abbey Road, St. John's Wood, of which I am honorary secretary, and we have kept her here hoping that she might recover; but as that is not the case, and we cannot give her the care and attention she requires, we are now seeking to place her where she will be happy and comfortable, and well taken care of. She seems to have no relatives or friends who can help her, and her means are very small. She would require a separate bedroom, and to be amongst ladies. She pays now, for her accommodation, including board, medical attendance, and medicine, 13*s*. 6*d*. per week, and I doubt if she could do any more. I have been advised to apply to you for information and help to know where to place her, and shall be greatly obliged for any information you can give me."

